# Association between preoperative haemoglobin concentration and cardiopulmonary exercise variables: a multicentre study

**DOI:** 10.1186/2047-0525-2-18

**Published:** 2013-09-13

**Authors:** James M Otto, Alasdair F O’Doherty, Philip J Hennis, Jackie A Cooper, Michael PW Grocott, Chris Snowdon, John B Carlisle, Michael Swart, Toby Richards, Hugh E Montgomery

**Affiliations:** 1 Division of Surgery and Interventional Science, University College London, 21 University Street, London WC1E 6DE, UK; 2 The Portex Unit, UCL Institute of Child Health, 30 Guilford Street, London WC1N 1EH, UK; 3 Department of Medicine, Centre of Cardiovascular Genetics, University College London, 5 University Street, London WC1E 6JF, UK; 4 Integrative Physiology and Critical Illness Group, Division of Clinical and Experimental Science, Faculty of Medicine, University of Southampton, University Road, Southampton SO17 1BJ, UK; 5 UCL Institute for Human Health and Performance, c/o 4th Floor, Rockefeller Building, 21 University Street, London WC1E 6DE, UK; 6 Newcastle Upon Tyne NHS Foundation Trust, Freeman Hospital, Freeman Road, High Heaton, Newcastle Upon Tyne NE7 7DN, UK; 7 Torbay Hospital, South Devon Healthcare NHS Foundation Trust, Torbay Hospital, Lawes Bridge, Torquay TQ2 7AA, UK

**Keywords:** Anaemia, Cardiopulmonary exercise testing, CPET, Haemoglobin concentration, Oxygen uptake, Surgery

## Abstract

**Background:**

Preoperative anaemia and low exertional oxygen uptake are both associated with greater postoperative morbidity and mortality. This study reports the association among haemoglobin concentration ([Hb]), peak oxygen uptake ($\dot{V}O_{2}$ peak) and anaerobic threshold (AT) in elective surgical patients.

**Methods:**

Between 1999 and 2011, preoperative [Hb] and cardiopulmonary exercise tests were recorded in 1,777 preoperative patients in four hospitals. The associations between [Hb], $\dot{V}O_{2}$ peak and AT were analysed by linear regression and covariance.

**Results:**

In 436 (24.5%) patients, [Hb] was <12 g dl^-1^ and, in 83 of these, <10 g dl^-1^. Both AT and $\dot{V}O_{2}$ peak rose modestly with increasing [Hb] (r^2^ = 0.24, *P* <0.0001 and r^2^ = 0.30, *P* <0.0001, respectively). After covariate adjustment, an increase in [Hb] of one standard deviation was associated with a 6.7 to 9.7% increase in $\dot{V}O_{2}$ peak, and a rise of 4.4 to 6.0% in AT. Haemoglobin concentration accounted for 9% and 6% of the variation in $\dot{V}O_{2}$ peak and AT respectively.

**Conclusions:**

To a modest extent, lower haemoglobin concentrations are independently associated with lower oxygen uptake during preoperative cardiopulmonary exercise testing. It is unknown whether this association is causative.

## Background

Increased mitochondrial oxygen uptake requires increased cellular oxygen delivery. When oxygen delivery, or utilization, fails to meet metabolic demand, anaerobic cytoplasmic metabolism significantly augments aerobic mitochondrial ATP generation with a consequent increase in lactic acid production and accumulation. It has been suggested that an imbalance in oxygen demand-supply contributes to the point during cardiopulmonary exercise testing (CPET) known as the anaerobic threshold (AT) [1], although this physiological underpinning is not without controversy [2,3]. Major surgery places substantial metabolic demands upon the patient and may increase resting oxygen uptake from an average preoperative value of 110 ml min^-1^ m^-2^ to approximately 170 ml min^-1^ m^-2^[4,5]. Lower preoperative exertional oxygen uptake ($\dot{V}O_{2}$), both the peak and that noted at AT, are associated with postoperative morbidity and mortality [6-8].

Each gram of haemoglobin carries 1.34 ml of oxygen when fully saturated. Anaemia, commonly defined as a haemoglobin concentration ([Hb]) below 13 g dl^-1^ (males) and 12 g dl^-1^ (non-pregnant females), reduces the blood’s oxygen carriage capacity. Anaemia is common amongst preoperative patients, with a prevalence ranging from 5% to 76% [9]. Anaemia and blood transfusion are associated with poor postoperative outcomes [10-16].

Oxygen delivery limits maximal oxygen uptake during exercise under normoxic conditions [17,18]. Increases in [Hb] increase $\dot{V}O_{2}$ peak, whilst acute reductions in [Hb] lower $\dot{V}O_{2}$ peak and endurance performance [19-21]. However, the extent to which postoperative outcomes are dependent upon interactions between [Hb] and $\dot{V}O_{2}$ is unknown. We analysed a large multicentre dataset to explore the relationship between preoperative [Hb] and $\dot{V}O_{2}$.

## Methods

### Patient population

We analysed cardiopulmonary exercise (CPET) data collected between December 1999 and February 2011 by four centres: University College London Hospitals NHS Trust (UCLH); the Whittington Hospital NHS Trust; Torbay Hospital, South Devon Healthcare NHS Foundation Trust; and the Freeman Hospital, Newcastle Upon Tyne Hospitals NHS Foundation Trust. Patients had been routinely tested as part of the clinical service before elective surgery: maxillofacial, hepatobiliary, vascular, upper gastrointestinal, colorectal, orthopaedic, bariatric and other specialties (mainly urological).

Following discussion among the researchers and the local Research and Development and clinical governance departments, formal ethical approval was waived and confirmation of audit status granted due to the nature of the data collection.

### Cardiopulmonary exercise testing

Across all testing sites, CPET was performed according to the American Thoracic Society/American College of Chest Physicians (ATS/ACCP) guidelines, under stable environmental conditions, with continuous 12 lead ECG monitoring and in the presence of a clinician [22].

Patients pedalled an electromagnetically-braked cycle ergometer (Lode BV, Groningen, Netherlands), with breath-by-breath respiratory gas analysis performed by various machines, (UCL-Cortex Biophysik, Leipzig, Germany; Torbay and Newcastle-Medical Graphics, Minnesota, USA) calibrated according to ATS/ACCP guidelines. During exercise, oxygen uptake ($\dot{V}O_{2}$) and carbon dioxide output ($\dot{V}CO_{2}$) were recorded, together with respiratory rate, tidal volume, ventilation and end-tidal gas tensions.

A 3-minute rest period followed fitting of relevant equipment, after which unloaded cycling was performed at a cadence of 60 to 70 rpm for 3 minutes. Thereafter, patients performed a symptom-limited continuous incremental exercise ramp protocol, determined by the physiologist or clinician on the basis of predictive work rate algorithms and patient-reported activity levels [23]. The test continued (usually for 8 to 12 minutes) until volitional exhaustion occurred, or the patient was unable to maintain a cadence of 40 rpm for more than 30 seconds despite encouragement. The clinician stopped the test if the patient developed a sign or symptom listed in the ATS/ACCP guidelines, which included: new arrhythmia; more than 2 mm of ST elevation or depression on the ECG; an arterial blood pressure of more than 250 mm Hg systolic or 120 mm Hg diastolic (see 2003 ATS/ACCP statement on Cardiopulmonary Exercise Testing for an exhaustive list) [23]. Following termination of CPET, patients were encouraged to perform a ‘warm-down’ period of unloaded cycling.

The anaerobic threshold was estimated by an exercise physiologist or consultant physician, both experienced in CPET interpretation, using a combination of the modified V-slope, ventilatory equivalents and end-tidal pressure methods [24], which improves the rigor of AT detection. The $\dot{V}O_{2}$ peak was recorded as the highest average $\dot{V}O_{2}$ over the final 30 s period [25]. The ventilatory equivalents for carbon dioxide ($\dot{V}E/\dot{V}CO_{2}$) and oxygen ($\dot{V}E/\dot{V}O_{2}$) were recorded at the AT [26].

We recorded age, sex, height, weight, body mass index (BMI, kg m^-2^) and Lee’s Revised Cardiac Risk Index (RCRI) from patients’ medical histories [27]. In addition, serum creatinine, obtained from hospital electronic records systems, was used as an index of renal function. At UCLH and the Whittington Hospital, [Hb] was measured on the day of CPET (HemoCue AB, Angelholm, Sweden). Preoperative [Hb] was recorded from hospital electronic record systems within 3 days of CPET at Newcastle and at the time of pre-assessment (usually within 4 weeks of CPET) at Torbay. No patient received a blood transfusion between the [Hb] measurement and the CPET.

### Statistical analysis

Statistical analysis was performed using Stata Version 11 (StataCorp, Texas, USA). Gaussian distributions of the data were verified by the Kolmogorov-Smirnov test, in conjunction with visual inspection of histogram charts. A difference between data was considered significant if *P* <0.05. We transformed seven continuous variables with skewed distributions by taking their log_10_: weight, BMI, AT, $\dot{V}O_{2}$ peak, $\dot{V}E/\dot{V}CO_{2}$, $\dot{V}E/\dot{V}O_{2}$, and creatinine. These variables are presented as geometric means and approximate SD.

Historically, measurements of oxygen uptake have been indexed to body mass (ml kg^-1^ min^-1^), as it allows comparisons between individuals [28,29]. However, this value may still vary with body mass [30-32]. We therefore adjusted the measured oxygen uptake by raising the body mass to a power determined by allometric scaling using the power function ratio (Y/X^β^) [29,33]. Specifically, the allometric relationship between body size and performance measure (AT or $\dot{V}O_{2}$ peak) is determined by the allometric equation below (see equation 1), where Y is AT or $\dot{V}O_{2}$ peak, X is body mass, β is a scaling exponent, a is the proportionality constant (intercept), and ϵ is the multiplicative error term, which overcomes the problem of heteroscedasticity [34].

(1) $$Y={\mathrm{aX}}^{\beta }\epsilon$$

The allometric relationship between body mass (X) and fitness parameter (Y) is expressed using the logarithmic transformation of equation 1 so that

(2) logY=β•logmass+loga+logϵ

where β is the sample specific slope of the linear least squares regression line calculated by log-linear regression analysis (that is, scaling exponent β was 0.83 in the current study) and log a is the equivalent constant value (a) [34]. We further built models by adjustment for the determinant variable (AT and $\dot{V}O_{2}$ peak) to potential confounders. Three levels of increasing adjustment were used: i) a basic adjustment for testing site; ii) an extended adjustment for testing site, age and sex; and iii) a fully adjusted model for all known confounders (testing site, age, sex, revised cardiac risk index, diabetes, creatinine and operation category). Results were standardised for testing centre by the inclusion of dummy variables in the regression model.

The effect size was expressed as the percentage increase in $\dot{V}O_{2}$ for a 1 g dl^-1^ (or one standard deviation) increase in [Hb]. Partial correlations between [Hb] and CPET markers were performed controlling for confounding variables. Regression models assessed the associations between $\dot{V}O_{2}$ and [Hb] and the proportion of variance in oxygen uptake explained by variation in [Hb]. Covariance models were analysed with [Hb] as a clinically relevant categorical variable ([Hb] <10 g dl^-1^; 10 to 12 g dl^-1^; >12 g dl^-1^), as similarly described [15,35]. The adjusted values for $\dot{V}O_{2}$ generated by the model were transformed back to the original scale to give geometric means and approximate standard deviations by [Hb] group.

## Results

We analysed data from 1,777 patients (1,108 male) undergoing various operations: 549 vascular (31%), 530 colorectal (30%), 337 bariatric (19%), 75 upper gastrointestinal (4%), 66 hepatobiliary (4%), 48 maxillofacial (3%) and 172 other operations (9%). Contributions from each centre were as follows: 804 UCLH; 484 Whittington; 305 Torbay; 184 Newcastle. The mean (SD) $\dot{V}O_{2}$ peak and AT were 15.5 (5.9) and 11.2 (3.5) ml kg^-1^ min^-1^ respectively. The $\dot{V}E/\dot{V}O_{2}$ and $\dot{V}E/\dot{V}CO_{2}$ at AT were 25.9 (6.4) and 30.8 (6.4). The AT was not identified in 146 patients (8.2%). Mean (SD) [Hb] and creatinine were 13.2 (1.8) g dl^-1^ and 82 (30) μmol l^-1^. Table 1 lists other physical characteristics.

**Table 1:** Physical characteristics of the whole-study cohort

	**N**	**Mean**	**SD**
Age (yr)	1,777	61.9	15.8
Height (cm)	1,776	169.0	9.1
Weight (kg)	1,775	83.7	23.6
BMI (kg m^-2^)	1,774	29.4	8.3
$\dot{V}O_{2}$ peak (ml min^-1^)	1,774	1300	500
AT (ml min^-1^)	1,631	940	300

*BMI* body mass index, *AT* anaerobic threshold, $\dot{V}O_{2}$ peak, peak oxygen uptake.

### Relationships between haemoglobin concentration and oxygen uptake

**Figure 1:**
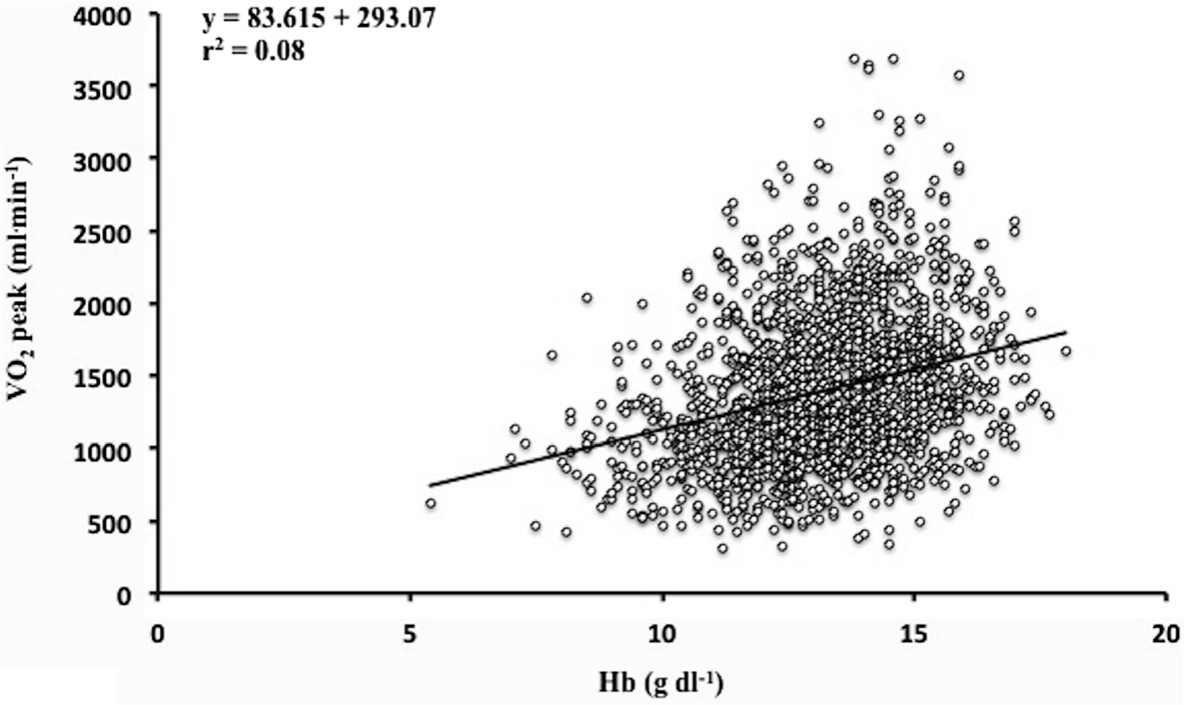
**Linear regression between unadjusted**$\dot{V}O_{2}$**peak (ml min**^**-1**^**) and haemoglobin concentration ([Hb]), n = 1,774.**

**Figure 2:**
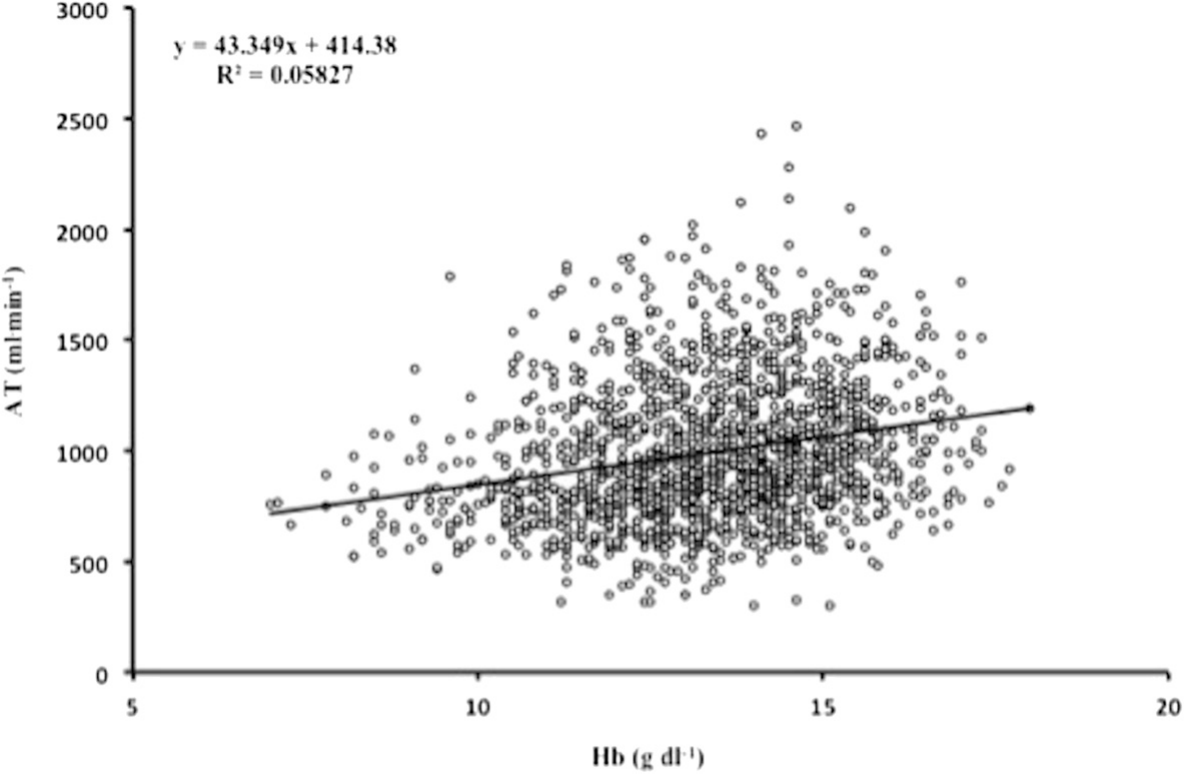
**Linear regression between unadjusted oxygen uptake at anaerobic threshold (AT) (ml min**^**-1**^**) and haemoglobin concentration ([Hb]), n = 1,631.**

Figure 1 graphs the increase in unadjusted $\dot{V}O_{2}$ peak with [Hb], whilst Figure 2 shows the relationship between unadjusted oxygen uptake at AT and [Hb]. The $\dot{V}O_{2}$ peak and AT increased across each [Hb] group (Table 2). More patients awaiting colorectal surgery were anaemic: in 144/530 (27%) of these, the [Hb] was 10 to 12 g dl^-1^ and in 32/530 (6%) the [Hb] was <10 g dl^-1^.

**Table 2:** **Physical characteristics across haemoglobin concentration ([Hb]) group (<10 g dl**^**-1**^**; Hb 10 to 12 g dl**^**-1**^**; Hb >12 g dl**^**-1**^**)**

	**[Hb]**		
	**<10 g dl**^**-1**^	**10 to 12 g dl**^**-1**^	**>12 g dl**^**-1**^
N	83	353	1341
Age (yr)	63.5 (16.8)	61.8 (16.4)	61.8 (15.5)
Height (cm)	165.8 (9.2)	166.1 (9.2)	170.0 (8.9)
Weight (kg)	73.3 (22.1)	78.9 (23.7)	85.7 (23.3)
BMI (kg m^-2^)	26.8 (8.1)	28.7 (8.7)	29.7 (8.2)
Creatinine (μmol l^-1^)	79.8 (39.0)	78.7 (29.5)	83.4 (28.9)
$\dot{V}O_{2}$ peak (ml min^-1^)^a^	960 (330)	1150 (430)	1380 (500)
$\dot{V}O_{2}$ peak (ml kg^-1^ min^-1^)^b^	13.0 (4.5)	14.5 (5.4)	16.1 (5.8)
$\dot{V}O_{2}$ peak (ml kg^-0.83^ min^-1^)^c, g^	27.8 (6.9)	31.5 (8.1)	34.3 (8.1)
AT (ml min^-1^)^d^	760 (190)	860 (260)	970 (310)
AT (ml kg^-1^ min^-1^)^e^	10.3 (2.5)	10.9 (3.2)	11.3 (3.6)
AT (ml kg^-0.83^ min^-1^)^f, g^	21.3 (4.3)	23.5 (4.8)	24.4 (4.8)
$\dot{V}E/\dot{V}O_{2}$	26.7 (4.8)	25.3 (5.4)	26.0 (6.8)
$\dot{V}E/\dot{V}CO_{2}$	31.7 (5.9)	30.2 (5.9)	31.0 (6.6)

*BMI* body mass index, $\dot{V}E/\dot{V}O_{2}$ and $\dot{V}E/\dot{V}CO_{2}$, ventilatory equivalents for oxygen and carbon dioxide at the AT; AT, anaerobic threshold; $\dot{V}O_{2}$ peak, peak oxygen uptake. Values are mean (SD). ^a^ANOVA, *P* <0.0001; linear trend, *P* <0.0001. ^b^ANOVA, *P* <0.0001; linear trend, *P* < 0.0001. ^c^ANOVA, *P* <0.0001; linear trend, *P* <0.0001; ^d^ANOVA, *P* <0.0001; ^e^linear trend, *P* < 0.0001; ^f^linear trend, *P* < 0.0001; ^g^Adjusted for weight, testing site, age, sex, revised cardiac risk index, diabetes, creatinine and operation category.

Haemoglobin concentration showed weak correlation with $\dot{V}O_{2}$ peak (r^2^ 0.30, *P* <0.0001) and AT (r^2^ 0.24, *P* <0.0001), after adjusting for weight and testing centre. Correlations between [Hb] and $\dot{V}O_{2}$ peak at each site were: Whittington (r^2^ 0.30, *P* <0.0001), Torbay (r^2^ 0.16, *P* = 0.004), UCLH (r^2^ 0.31, *P* <0.0001), and Newcastle (r^2^ 0.33, *P* <0.0001). Correlations between [Hb] and AT at each site were; Whittington (r^2^ 0.23, *P* <0.0001), Torbay (r^2^ 0.23, *P* <0.0001), UCLH (r^2^ 0.24, *P* <0.0001), and Newcastle (r^2^ 0.33, *P* <0.0001).

### Regression models

An increase in [Hb] by one SD was associated with a 9.7% (95% CI, 8.2 to 11.3) increase in $\dot{V}O_{2}$ peak after adjusting for weight (*P* <0.0001), which was reduced to 6.7% (95% CI, 5.4 to 7.8) after adjusting for age, sex, weight and testing centre (*P* <0.0001). The percentage of the variance in $\dot{V}O_{2}$ peak explained by [Hb] was 8.9% (*P* <0.0001) after adjusting for weight, and 5.5% (*P* <0.0001) after adjusting for age, sex, weight and testing site. An increase in [Hb] by one SD was associated with a 6.0% (95% CI, 4.8 to 7.3) increase in AT after adjusting for weight *P* <0.0001), which was reduced to 4.4% (95% CI, 3.3 to 5.5) after adjusting for age, sex, weight and testing centre (*P* <0.0001). The percentage of variance in AT explained by [Hb] was 5.9% (*P* 0.0001) after weight adjustment, reducing to 3.5% (*P* <0.0001) after adjusting for age, sex, weight and testing centre.

### Analysis of covariance across haemoglobin group

**Figure 3:**
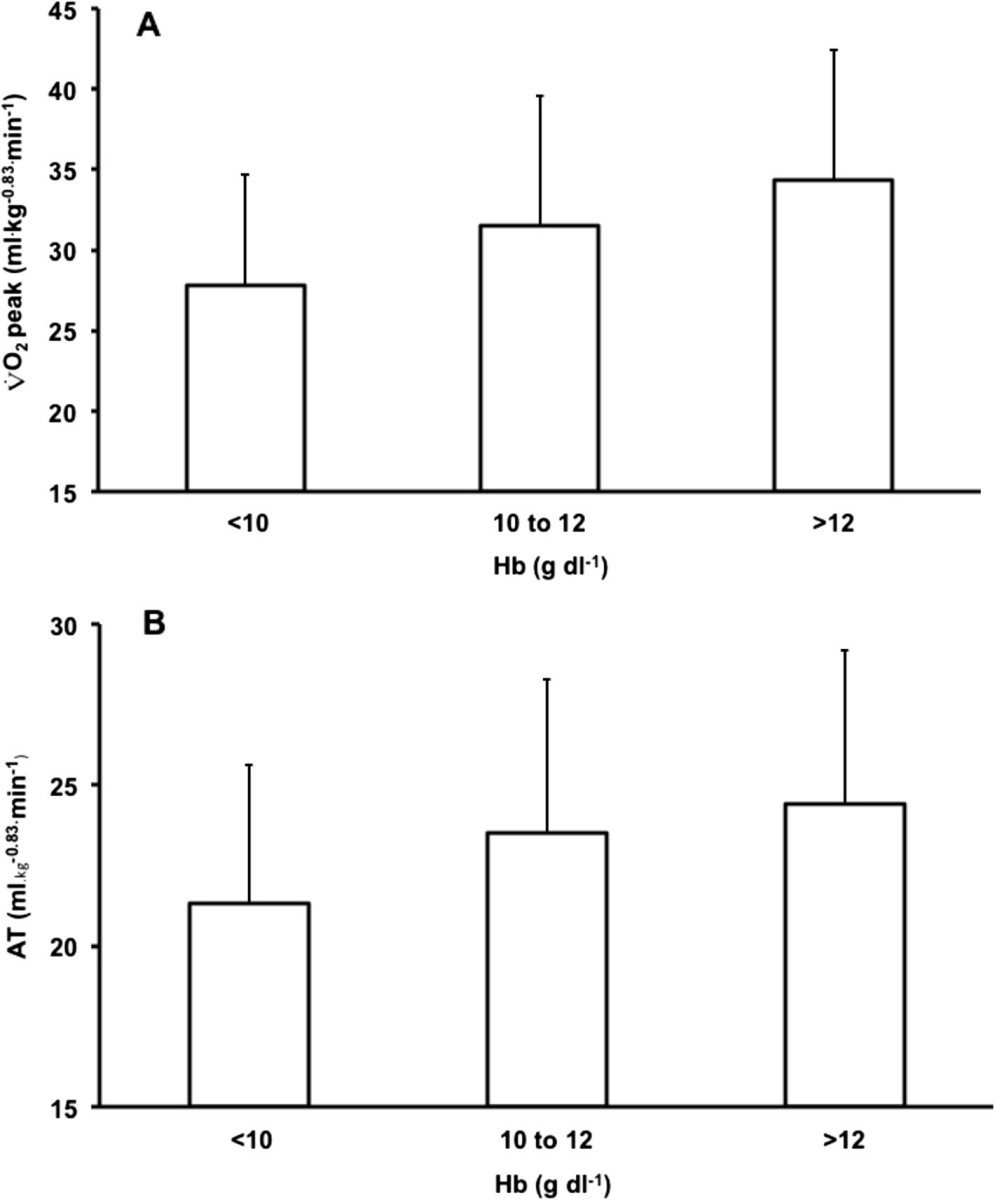
**Allometrically scaled oxygen uptake (ml kg**^**-0.83 **^**min**^**-1**^**) across [Hb] group (<10 g dl**^**-1**^**, 10 to 12 g dl**^**-1**^**, >12 g dl**^**-1**^**);**$\dot{V}O_{2}$**peak (A) and at the anaerobic threshold (AT) (B).** Mean +/- SD values adjusted for weight, testing site, age, sex, revised cardiac risk index, diabetes, creatinine and operation category. *P* <0.0001 (ANOVA) and trend (*P* <0.0001) for both measures. Hb, haemoglobin concentration.

$\dot{V}O_{2}$ peak and AT increased across [Hb] groups, after adjusting for confounding variables (*P* <0.0001, Table 3 and Figure 3).

**Table 3:** Allometrically scaled oxygen uptake

	**Haemoglobin concentration**			***P*-value**	
	**<10 g dl**^**-1**^	**10 to 12 g dl**^**-1**^	**>12 g dl**^**-1**^	**ANOVA**	**Trend**
$\dot{V}O_{2}$ peak (ml kg^-0.83.^min^-1^)	26.7 (7.8) 83	29.9 (8.4) 352	34.4 (10.1) 1339	<0.0001	<0.0001^a^
	27.8 (7.1) 83	31.2 (8.2) 352	34.0 (8.7) 1333	<0.0001	<0.0001^b^
	27.8 (6.9) 64	31.5 (8.1) 291	34.3 (8.1) 1136	<0.0001	<0.0001^c^
AT (ml kg^-0.83.^min^-1^)	20.9 (4.6) 67	22.7 (5.0) 307	24.4 (5.2) 1257	<0.0001	<0.0001^a^
	21.3 (4.5) 67	23.2 (4.9) 307	24.3 (5.1) 1251	<0.0001	<0.0001^b^
	21.3 (4.3) 49	23.5 (4.8) 252	24.4 (4.8) 1068	<0.0001	<0.0001^c^

$\dot{V}O_{2}$ peak and AT, mean (SD) number of patients, by haemoglobin classification. *AT* anaerobic threshold, *[Hb]* haemoglobin concentration, $\dot{V}O_{2}$ peak, peak oxygen uptake.

^a^Adjusted for weight and testing site.

^b^Adjusted for weight, testing site, age, sex.

^c^Adjusted for weight, testing site, age, sex, revised cardiac risk index, diabetes, creatinine and operation category.

We analysed allometrically scaled $\dot{V}O_{2}$ peak across individual surgical cohorts, with and without adjustment for confounders. $\dot{V}O_{2}$ peak did not differ in hepatobiliary patients across [Hb] groups. $\dot{V}O_{2}$ peak increased across each [Hb] group for colorectal, bariatric and other (mainly urological) patients, after adjusting for all confounders (testing centre, age, sex, weight, RCRI, diabetes, serum creatinine and operation category). $\dot{V}O_{2}$ peak increased across each [Hb] classification for upper gastrointestinal and vascular patients after adjustment for testing centre, age, sex and weight, but not when additional confounders (RCRI, diabetes, serum creatinine and operation category) were added. $\dot{V}O_{2}$ peak increased with each [Hb] group for maxillofacial patients after adjusting for testing and weight, but not when additional confounders were added to the model.

## Discussion

To our knowledge, this is the largest study to explore the relationship between oxygen uptake and haemoglobin concentration in the clinical setting, and the first to control for body mass with allometric (log-linear) scaling. Oxygen uptake at peak exercise and at the anaerobic threshold (AT) increased with haemoglobin concentration [Hb], after adjusting for measured confounding variables.

The [Hb] was 10 to 12 g dl^-1^ in 353 patients (20%) and <10 g dl^-1^ in 83 (5%) patients. Other studies have reported rates of anaemia between 5% and 76%, a range partly dependent upon the indication for surgery and definition of anaemia [9]. The American College of Surgeons’ National Surgical Quality Improvement Program (ACS NSQIP) recently reported a similar prevalence of preoperative anaemia (30.4%) [15].

The mean AT of 11 ml kg^-1^ min^-1^ is similar to that reported by other studies of preoperative populations [7,36,37]. However, this value is 2 to 3 ml kg^-1^ min^-1^ less than gender-specific reference values for age and height [38]. The mean $\dot{V}O_{2}$ peak in our population, 15.5 ml kg^-1^ min^-1^, is 11 ml kg^-1^ min^-1^ less than gender-specific reference values for age and height [38,39]. The aim of allometric scaling is to appropriately account for body size (that is, the scaled variable no longer varies with body size) [40]. Oxygen uptake is usually reported per unit body mass, ml kg^-1^ min^-1^, a scale that requires further adjustment for body size [41]. In the obese, oxygen uptake expressed as ml kg^-1^ min^-1^ underestimates fitness and overestimates risk [42]. In the cachectic patient this scale overestimates fitness and underestimates risk [43].

Both AT and $\dot{V}O_{2}$ peak increased with [Hb], for the whole population, across individual testing sites and across all groups, except hepatobiliary surgery. However, this relationship was weak and although being highly statistically significant does not necessarily reflect a magnitude of clinical association. Nonetheless, an increase in [Hb] by one standard deviation (that is, 1.8 g dl^-1^ rise in [Hb]) was associated with a 9.7% and 6.0% increase in weight-adjusted $\dot{V}O_{2}$ peak and AT. The [Hb] explained 9% and 6% of the variance in $\dot{V}O_{2}$ peak and AT respectively. The increase in AT and $\dot{V}O_{2}$ peak with [Hb] may be due to increased oxygen-carrying capacity, or patients who are not anaemic exercising more than patients who are anaemic, or due to confounding. For instance, sick patients may be both anaemic and less fit. In addition, differences in AT, to some extent (although probably small), may be explained by inherent variations in measurement and/or interpretation or physiological context [44].

The cause of anaemia may be important. The most common cause is reported to be chronic disease, the severity of which being related to the degree of systemic inflammation [45,46]. Features of anaemia due to chronic disease include reduced red cell survival, impaired erythropoiesis, and impaired iron metabolism, all of which may reduce AT and $\dot{V}O_{2}$ peak, directly or in combination [47]. Iron status was not routinely assessed in our cohort but may independently influence fitness markers in the absence of anaemia. For example, iron deficiency with or without anaemia is associated with reduced fitness [48-50].

This study had some limitations. The observational, cross-sectional and retrospective nature of the data generates causative hypotheses but does not test them [51]. It would have been valuable to assess the association of overall survival and critical care use with both anaemia and oxygen uptake. The [Hb] may be an imprecise measure of blood oxygen carrying capacity, given that its value is influenced by disease- or therapy-related contractions or expansions in plasma volume. For instance, oxygen-carrying capacity may be normal if [Hb] is low due simply to an increase in intravascular volume. A better measure of oxygen-carrying capacity may thus be total mass of haemoglobin (tHb-mass). The tHb-mass displays a higher correlation with $\dot{V}O_{2}$ peak (r^2^ = 0.79) than either blood volume (r^2^ = 0.76) or [Hb] [52,53]. The relatively small explained variance in AT and $\dot{V}O_{2}$ peak by [Hb] (oxygen carrying capacity) in the current study suggests that other factors may play an important role in determining aerobic capacity. For example, other physiological factors that may limit $\dot{V}O_{2}$ peak include pulmonary diffusing capacity, cardiac output and skeletal muscle limitations [54]. Although it is suggested that the AT reflects an imbalance in oxygen demand-supply, that is, that AT reflects onset of anaerobiosis, this is a much-debated and controversial concept [44,55]. In addition, variations in the AT, to some extent may be explained by inherent discrepancies in its measurement and/or interpretation [44].

Future studies of preoperative cardiopulmonary exercise testing should include [Hb] with long-term survival and quality of life as outcomes as well as considering alternative endpoints measured during exercise testing such as metabolic efficiency and the oxygen pulse [44].

## Conclusions

In conclusion, anaemia is common in preoperative patients undergoing elective major surgery. There is an association between haemoglobin concentration and oxygen uptake during exercise, both $\dot{V}O_{2}$ peak and AT, even after adjusting for measured confounding variables. Future studies may wish to address whether reversing anaemia before surgery improves these values, and thereby increases postoperative survival and function.

## Abbreviations

AT: Anaerobic threshold; BMI: Body mass index; CPET: Cardiopulmonary exercise testing; [Hb]: Hemoglobin concentration; RCRI: Revised cardiac risk index; tHb-mass: Total haemoglobin mass; $\dot{V}CO_{2}$: Carbon dioxide; $\dot{V}E/\dot{V}O_{2}$: Ventilatory equivalent for oxygen; $\dot{V}E/\dot{V}CO_{2}$: Ventilatory equivalent for carbon dioxide; $\dot{V}O_{2}$: Oxygen uptake; $\dot{V}O_{2}$ peak: peak oxygen uptake.

## Competing interests

JM Otto: JMO is receiving an Impact PhD Studentship part-funded by VIFOR (INTERNATIONAL) Inc. Total funding £32,534 over 3 years.

MPW Grocott: MPWG has received honoraria for speaking and / or travel expenses from: Edwards Lifescience, Fresenius-Kabi, BOC Medical (Linde Group), Ely-Lilly Critical Care, Cortex GmBH.

MPWG has received research grants from: National Institute of Health Research, National Institute of Academic Anaesthesia, Intensive Care Society, Association of Anaesthetists of Great Britain and Ireland, Sir Halley Stuart Trust, Francis and Augustus Newman Foundation. MPWG is the R&D Lead for Division A, University Hospitals Southampton NHS Foundation Trust; Director, National Institute of Academic Anaesthesia Health Services Research Centre; Specialty Group Lead (Critical Care and Anaesthesia), Hampshire and Isle of Wight Comprehensive Local Research Network, NIHR Comprehensive Research Network.

MPWG leads the Xtreme-Everest hypoxia research consortium and the group have received unrestricted research grant funding from: BOC Medical (Linde Group), Ely-Lilly Critical Care, Smiths Medical, Deltex Medical, London Clinic, Rolex. MPWG runs a number of educational meetings and these meetings have sponsorship from multiple industry partners declared on a meeting-bymeeting basis.

MPWG: Board and Research Council member of the National Institute of Academic Anaesthesia, Co-Chairman of Evidence Based Perioperative Medicine (annual scientific meeting), Co-Chairman of Current Controversies in Anaesthesia and Perioperative Medicine (annual scientific meeting), Co-chairman of National Perioperative CPET Meeting (annual scientific meeting), Co-chairman of KnO2wledge (annual scientific meeting), Member organising group, UK Perioperative Clinical Research Forum (annual scientific meeting), Faculty of Perioperative CPET course, Executive faculty of UK-UIAA Diploma in Mountain Medicine, Editor in Chief of Extreme Physiology and Medicine, Editorial board of Perioperative Medicine and British Journal of Hospital Medicine, Member of the Improving Surgical Outcomes Group. All remaining authors declare that they have no competing interests.

## Authors’ contributions

JMO, AFO, PJH, CS, JBC, and MS were responsible for data collection, drafting and revising the article and final approval of the version to be published. JAC was responsible for statistical analyses, revising the article and final approval of the version to be published. MPWG was responsible for substantial contribution to conception and design and facilitating acquisition of data, revising the article and final approval of the version to be published. TR and HEM were responsible for substantial contribution to conception and design, revising the article and final approval of the version to be published. All authors read and approved the final manuscript.
